# Emerging resistance of *van* genotype in enterococci: A potential menace for therapeutic failure

**DOI:** 10.12669/pjms.35.6.1145

**Published:** 2019

**Authors:** Hasan Ejaz

**Affiliations:** Dr. Hasan Ejaz, PhD, Department of Clinical Laboratory Sciences, CAMS, Jouf University, Sakaka, Saudi Arabia

**Keywords:** *E. faecalis*, *E. faecium*, Glycopeptide resistance, Linezolid, Vancomycin resistant enterococci, *van* genotypes

## Abstract

**Objectives:**

Emerging cases of vancomycin-resistant enterococci (VRE) are detrimental for the patients. The current study aimed to ascertain the occurrence of VRE, their antibiogram and the *van* genotype responsible for vancomycin resistance.

**Methods:**

A total number of 2,958 clinical specimens were processed at Microbiology Department of the Alrazi Health Care, Lahore during the one year (2016-2017) using microbiological culture media, biochemical and serology. Antibiogram of enterococcal strains was performed using disc diffusion and E-test. ATCC *Enterococcus faecalis* 29212 was used as a quality control strain. The detection of *van* genotypes was accomplished by multiplex PCR assay.

**Results:**

Out of the 147 enterococci, 139 (94.6%) were *E. faecalis*, and 8 (5.4%) were *E. faecium*. Statistically significant associations of urine (p < 0.001), pus (p < 0.001) and wound swabs (p = 0.001) were observed with *E. faecalis*. A significant correlation of enterococcal infections (p = 0.05) was seen with female patients. Four (2.9%) strains of *E. faecalis* found to be VRE with *vanB* (75%) and *vanA* (25%) genotypes.

**Conclusion:**

The emerging strains of VRE (*vanB* and *vanA* genotype) in the current study are a potential menace for therapeutic failure, which left the physicians with only linezolid as a therapeutic option.

## INTRODUCTION

*Enterococcus* species were considered to be harmless and unimportant to humans medically many years ago. They have the ability to produce bacteriocins, probiotic and have been used extensively in the food industry.^1^ Medically, they are opportunistic, lactic acid Gram-positive cocci which are mainly residents of gastrointestinal (GI) tract of humans and animals but now they have been contributed approximately 80-90% of human enterococcal infections in which *E. faecalis* and *E. faecium* play the major part.^2^ They have emerged as a significant part of nosocomial pathogens among others and are a reason for increased rate of mortality.^3^ Multidrug-resistant enterococcal infections should be given the same significance as methicillin-resistant *Staphylococcal* infections and the infections caused by extended-spectrum beta-lactamase-producing bacterial strains.^4^-^6^

More than 17 species of genus *Enterococcus* have been discovered, but *E. faecalis* and *E. faecium* are the predominating. Some other species (*E. avium, E. gallinarum* and *E. casseliflavus*) are also accountable for infections in humans.^7^ Enterococci can cause a variety of infections which can be life-threating such as endocarditis bacteremia, intravenous catheter infection and UTI.^8^ They are multidrug resistance against extensive classes of drugs that include beta-lactams and aminoglycosides and have left only few treatment options for clinicians. The acquisition of resistance is accomplished by transposons, plasmids and chromosomal transfer or mutations against different groups of antibiotics. From the past two decades, enterococci have acquired resistance against many commonly used drugs and their potential for gaining and spreading resistant genes with ease is alarming.^9^

The vancomycin resistance amongst the enterococcal species identified in the 1980s in England. A pronounced increase has been found in the popularity of the vancomycin resistance, which is a glycopeptide antibiotic.^10^ Diverse types of vancomycin-resistant genes have been identified, which is worrisome and keep a significant concern. Enterococcal glycopeptide resistance can be mediated by the *vanA* (vancomycin and teicoplanin resistance), *vanB* genes (vancomycin resistance) and *vanM* (vancomycin and teicoplanin resistance) genes.^11^ The rest of the *van* genes responsible for the glycopeptide resistance are *vanC, vanD, vanE, vanG, vanL* and *vanN*.^12^ Prompt and precise detection of vancomycin-resistant enterococci (VRE) is necessary to reduce the morbidity among the infected patients which is usually done by the molecular methods.^13^ The current study aimed to ascertain the prevalence of VRE in various clinical sources, their antibiogram and genetic basis of vancomycin (*van* genotype) resistance.

## METHODS

The bacterial strains for this cross-sectional study were collected from the Microbiology Department of the Alrazi Health Care Lahore, Pakistan, over twelve months from July 2016 to June 2017 after IRB approval (Ref.no. 13A/ARHC, Date on: June 15, 2016). A total number of 2,958 various clinical samples of urine, pus, wound swabs, ear swabs, endotracheal tubes (ETT), blood, cerebrospinal fluid (CSF) and other body fluids were processed to detect the occurrence of enterococci consecutively. Samples were processed to isolate enterococci from both genders of different age groups.

The clinical specimens were inoculated on different culture media (Blood, Chocolate, MacConkey’s and CLED) and incubated at 37°C for overnight.^14^ Enterococci were identified by physical characteristics, Gram’s reaction, biochemical (catalase reaction) and serological testing (reaction to Group-D antisera). Moreover, the tolerance of enterococci was checked by 6.5% NaCl and the ability to grow on bile esculin agar. The non-enterococcal concomitant organisms were excluded from the study.

Enterococcal antibiogram was performed by using Kirby Bauer disc diffusion method, and bacterial suspensions were made according to 0.5 McFarland’s scale by streaking the bacterial suspension on the surface of the blood agar plate. The antibiotic discs of penicillin (10 U), ampicillin (10 µg), co-amoxiclav (20/10 µg), ceftazidime (30 µg), linezolid (30 µg), ceftriaxone (30 µg), cefuroxime (30 µg), piperacillin-tazobactam (100/10 µg), clindamycin (10 µg) and erythromycin (15 µg) were used to observe the antibiogram. E-test strips (Liofilchem, Italy) were used to determine the minimum inhibitory concentration of vancomycin. After 24 hours’ incubation at 35-37^o^C, results of these drugs were recorded as mentioned in the Clinical and Laboratory Standard Institute (CLSI) manual. ATCC *E. faecalis* 29212 was used as a quality control strain. Results of each isolate were reported as sensitive, intermediate sensitive or resistant to the antimicrobial disc based on the interpretation chart of zone sizes recommended by CLSI.^15^

The detection of *vanA, vanB, vanC1* and *vanC2/C3* was done using multiplex PCR assay. The previously mentioned primers were used for the detection of *vanA, vanB, vanC1* and *vanC2/C3*.^16^ The DNA was extracted in 100 µl TE buffer using boiling method for 10 minutes. The PCR conditions were optimised using different gradient temperatures for annealing and finally adjusted as initial denaturation (95°C, 10 minutes) followed by 29 cycles of denaturation (95°C, 1 minute), annealing (55°C, 50 seconds), DNA extension (72°C, 55 seconds) and final extension at 72°C for 10 minutes. Multiplex PCR products were analysed using 1% Agarose gel, SYBR Safe (gel staining dye) and 100 bp molecular ruler. No template control was also included with each run of electrophoresis. Chi-square test used to analyze the p-values for the statistical analysis using SPSS v.24.

## RESULTS

A total of 147 enterococcal strains were isolated from 2,958 different clinical isolates, amongst which 139 (94.6%) were *E. faecalis* and eight (5.4%) were *E. faecium*. Primarily, the sources of both of the enterococci were urine, pus and wound swabs. Statistically significant associations of urine (p < 0.001), pus (p < 0.001) and wound swabs (p = 0.001) were observed with *E. faecalis*. No statistically significant relationship of *E. faecalis* or *E. faecium* found with the other sources. Overall, the frequency of *E. faecalis* was higher in female patients (n=92; 62.5%) than male patients (n=47; 32%). Similarly, the frequency the *E. faecium* was higher among the female patients (n=6; 4.1%) than male patients (n=2; 1.4%). A borderline significant correlation of enterococcal infections (p = 0.05) was seen with female patients. The correlation of the patients with different age groups in the case of *E. faecalis* and *E. faecium* infections was not statistically significant (p = 0.85). The mean patient age ± SD in case if *E. faecalis* was 57.5 ± 24.6 while the mean age ± SD in case of *E. faecium* was 54.2 ± 16.5 (Table-I).

**Table I:** Source, age and gender distribution of cases infected with enterococci (n=147).

Specimens	Total cases n (%)	E. faecalis n (%)	E. faecium n (%)	p-value
Urine	1244 (42.1)	94 (63.9)	5 (3.4)	< 0.001
Pus	734 (24.8)	26 (17.7)	2 (1.4)	< 0.001
Wound Swab	275 (9.3)	13 (8.8)	1 (0.7)	0.001
Ear Swab	172 (5.8)	3 (2)	0	-
ETT Tips	19 (0.6)	2 (1.4)	0	-
Blood	452 (15.3)	1 (0.7)	0	-
Other Body Fluids	50 (1.7)	0	0	-
CSF	12 (0.4)	0	0	-

Total	n=2958	139 (94.6)	8 (5.4)	

***Gender [Enterococcal cases; n (%)]***				
Males	49 (33.3)	47 (32)	2 (1.4)	0.05
Females	98 (66.7)	92 (62.5)	6 (4.1)
***Age Groups [Enterococcal cases; n (%)]***				
Mean age ± S.D.		57.5 ± 24.6	54.2 ± 16.5	
0-20 years	18 (12.2)	18 (12.2)	0	0.85
21-40 years	60 (40.8)	57 (38.8)	3 (2)	
41-60 years	58 (39.5)	53 (36.1)	5 (3.4)	
61-80 years	11 (7.5)	11 (7.5)	0	

Total	147	139 (94.6)	8 (5.4)	

In vitro antibiogram of enterococci was observed against the various groups of antibiotics. All the isolated enterococcal strains were resistant to penicillin. There were 109 (78.4%) strains *E. faecalis* resistant to ampicillin, 99 (71.2%) to ceftriaxone, 98 (70.5%) to piperacillin-tazobactam, 90 (64.7%) to cefuroxime and 75 (54%) to ciprofloxacin. There were four (2.9%) strains *E. faecalis* found to be resistant to vancomycin. The resistance profile of *E. faecium* against different antibiotics was similar to *E. faecalis* with the very high resistance to ampicillin in seven (87.5%) cases, three (37.5%) to piperacillin-tazobactam, five (62.5%) to each of the ceftriaxone and cefotaxime. There was no case of vancomycin resistance detected with *E. faecium*. *E. faecalis* or *E. faecium* strains showed no resistance against linezolid (Table-II).

**Table II:** Antibiogram of enterococci against various antibacterial drugs.

Name of Antibiotic	E. faecalis (n=139) n (%)	E. faecium (n=8) n (%)
Penicillin	139 (100)	8 (100)
Ampicillin	109 (78.4)	7 (87.5)
Ceftriaxone	99 (71.2)	5 (62.5)
Piperacillin-tazobactam	98 (70.5)	3 (37.5)
Cefuroxime	90 (64.7)	5 (62.5)
Ciprofloxacin	75 (54)	4 (50)
Clindamycin	40 (28.8)	2 (25)
Erythromycin	37 (26.6)	0
Vancomycin	4 (2.9)	0
Linezolid	0	0

The vancomycin-resistant strains of *E. faecalis* were found to be positive for two *van* genotypes. In the molecular analysis, *vanA* genotype was detected in one (25%) isolate while *vanB* genotype was detected in three (75%) cases. None of the other genotypes detected in any of the isolates (Table-III).

**Table III:** Distribution of van genotypes among VRE (n=4).

Genotypes	E. faecalis n (%)	E. faecium n (%)
vanA	1 (25)	0
vanB	3 (75)	0
vanC1	0	0
vanC2/C3	0	0

## DISCUSSION

Enterococci constituted the healthy flora of gut of human and many animals, but now they have become an evident reason for nosocomial urinary infections and skin infections. *Enterococci* have been identified as a second known cause of nosocomial infections in US hospitals.^17^ The current study recorded a significant occurrence of enterococci in urine, pus and wound swabs. The number of *E. faecalis* (94.6%) was higher than the *E. faecium* (5.4%). A different prevalence of *E. faecalis* (62.5%) and *E. faecium* (23.9%) and other enterococci (13.6%) has also been reported.^18^ A study from Pakistan reported urine samples as a major source for the isolation of enterococci.^14^ The higher occurrence of enterococci had been reported as 43% from pus and 31% from the urine specimens with the distribution of 76% *E. faecalis* and 24% *E. faecium* which is slightly higher than the current study.^4^ The nosocomial enterococcal infections frequently tend to occur in surgical wounds and urinary tract of the patients. *E. faecalis* (85.7%) found to be the major organism followed by the second prevalent organism *E. faecium* (14.3%).^17^ The isolation of enterococci has also been reported other than these two sources, which predominantly include stool, sputum and throat swabs. *E. faecalis* (92%) remained to be higher in frequency than the *E. faecium* (8%), and these findings are close to the current study.^19^

The majority of the cases were between the age of 21-40 (40.8%) and 41-60 (39.5%) years and the enterococcal strains were isolated significantly higher from female patients. The occurrence of enterococci has been reported higher in female patients with a higher infection rate in 21-40 years.^19^ The greater occurrence of enterococci in 50-60 years of age has been reported by another study.^20^

Enterococci have become resistant to most of the β-lactam antibiotics, aminoglycosides, and glycopeptide (vancomycin), which made them an important nosocomial pathogen. In this study, enterococci found to be highly resistant to penicillin, cephalosporin and other classes of antibiotics with the emergence of 2.9% as VRE. The only choice remained to treat the VRE in this study was linezolid. A higher number of VRE (16.1%) with a similar resistance profile have been reported from a tertiary care paediatric hospital of Pakistan and linezolid remained the last option for the treatment of VRE.^14^ In an Indian study, the VRE found to be prevalent in 36% of the cases with the variable resistance to the different classes of antibiotics. The resistance against the ciprofloxacin (58%), piperacillin (54%), tetracycline (47%) and amoxicillin (47%) remained high. Linezolid and tigecycline were reported as the treatment of choice to treat VRE infections.^19^ The difference in the vancomycin resistance with other studies could be due to the walk-in cases in the current study. The hospitalised patients are more susceptible to the infections by the vancomycin-resistant strains as a result of nosocomial infections. The injudicious use of cephalosporin, ticarcillin, piperacillin-tazobactam and even vancomycin antibiotics helps in the emergence of VRE. These antibiotics reach to a higher gastrointestinal concentration with less activity against the enterococci.^21^

The *van* genotype responsible for the emergence of VRE in the current study was *vanB* (75%) and *vanA* (25%) which were detected in *E. faecalis* strains. The predominant genotype responsible for the appearance of resistance against vancomycin was found to be *vanA* followed by *vanC*.^18^ VRE have most commonly *vanA* and *vanB* genotype, but rarely an unusual genotype *vanM* can be detected.^22^ A similar prevalence of *vanA* and vanB among the VRE have been reported by another study using rapid PCR test.^23^ These results support the findings of the current study where the existence of only *vanB* and *vanA* found in the region. The existence of *vanA* has been reported in > 95% cases in some studies while a study also reported 3% of cases of *vanC1* genotype.^24^,^25^

### Limitations of the study

Due to limited resources, detection of other *van* genotypes could not be performed. The disease outcome and clinical data could not be managed for walk-in patients.

## CONCLUSION

*E. faecalis* and *E. faecium* constituted as the predominant strains among enterococci which are responsible for the nosocomial multidrug-resistant infection. A shift in the pattern of antibiotic resistance in enterococci has been observed, which is mediated by self-transferable plasmids with a broader host range. The emerging strains of VRE in the current study are a potential menace for therapeutic failure, which left the physicians with only linezolid as a therapeutic option. This study reports the emerging *vanB* and *vanA* multidrug-resistant genotype, which is worrisome. Prompt action is required to regulate the consumptions of antibiotics (particularly vancomycin), and implementation of effective infection control strategies, surveillance, screening procedures and preventive measures should be practised worldwide.
